# Polysaccharide extracted from *WuGuChong* reduces high-fat diet-induced obesity in mice by regulating the composition of intestinal microbiota

**DOI:** 10.1186/s12986-020-00442-2

**Published:** 2020-03-30

**Authors:** Wendong Wang, Mintao Zhong, Tiantian Yu, Lei Chen, Lijun Shi, Junwei Zong, Shouyu Wang

**Affiliations:** 1 Department of Orthopedic Surgery, The First Affiliated Hospital of Dalian Medical University, 222 Zhongshan Road, Dalian, China; 2 College of Integrative Medicine, Dalian Medical University, 9 South Lushun Road West, Dalian, China; 3 Department of Microbiology, Dalian Medical University, 9 South Lushun Road West, Dalian, China; 4 Department of Gynaecology and Obstetrics, Dalian Municipal Woman and Children’s Medical Center, 1 and 3 Sports new town first Road, Dalian, China

**Keywords:** Gut microbiota, High-fat diet, Obesity, Polysaccharides, *WuGuChong*

## Abstract

**Background:**

Obesity is a severe public health threat worldwide. Emerging evidence suggests that gut microbiota dysbiosis is closely associated with obesity and its related metabolic complications. The aim of the present study is to investigate the effects of polysaccharide extracted from *WuGuChong* (PEW) on high-fat diet (HFD)-induced obesity, and the potential mechanisms involving modulation of the gut microbiota composition.

**Methods:**

Mice were fed a normal chow diet and a high-fat diet with or without PEW (300 mg/kg/day) by oral gavage for 8 weeks. Body weight, obesity-related metabolic disorders, and gut microbiota were examined at the end of the experiment.

**Results:**

PEW supplementation reduces body weight, adipose hypertrophy, liver steatosis, insulin resistance and systemic inflammation in HFD-fed mice, as well as maintains intestinal epithelium integrity. High-throughput 16S rRNA analysis demonstrates that PEW supplementation alters the composition of gut microbiota. The *Firmicutes* to *Bacteroidetes* ratio and the relative abundance of *Proteobacteria* are increased in HFD-fed mice, which are reversed by PEW supplementation to approximately the control levels.

**Conclusions:**

Our results suggest that PEW may be used as a bioactive ingredient to prevent obesity and its related metabolic disorders by modulating the composition of gut microbiota.

## Background

Obesity is a global public health issue, and the major causes of obesity are considered to be unhealthy dietary patterns and lifestyles [1, 2]. Accumulating evidence suggests that obesity, which is closely related to chronic systemic inflammation and insulin resistance, is a risk factor for cancers and some other chronic diseases such as diabetes, atherosclerosis, fatty liver diseases [3–5]. High prevalence of obesity causes great damage to public health, however, many obesity-related therapeutic approaches, such as lifestyle changes, bariatric surgery, and pharmacotherapy, lead to an increased risk of various chronic diseases [6]. Finding novel therapeutic strategies is urgently necessary and challenging for prevention of obesity.

Increasing data indicates that gut microbiota composition is associated with obesity and its related metabolic disorders [7]. The gut microbiota composition varies significantly among obese and lean people [8]. For instance, an increase in the *Firmicutes*/*Bacteroidetes* (F/B) ratio can promote the development of obesity [9, 10]. Germ-free mice with gut microbiota transplanted from high fat diet (HFD)-induced obese donors exhibited significantly increased body weight gain and metabolic syndromes [11]. In HFD-induced obese models, gut microbiota is also closely linked with intestinal permeability, which is associated with gut integrity and barrier function [12]. Meanwhile, gut microbiota plays an important role in nutrient acquisition, energy harvest and lipid metabolism, which are related to metabolic diseases [7, 13, 14]. Endotoxins, bile acids and short-chain fatty acids, produced by gut microbiota, link gut microbiota to metabolic health [15–18]. Therefore, modulating the composition of gut microbiota may be a potential new strategy for preventing obesity and its related metabolic disorders.

Recently, accumulating evidence has demonstrated that some bioactive ingredients, especially polysaccharides, can reduce obesity by modulating the composition of the gut microbiota [9, 19]. For example, supplementation with polysaccharides isolated from sea cucumber or *Ganoderma lucidum*, reduced body weight gain and attenuated obesity-related metabolic syndromes in association with modulation of gut microbiota in HFD-fed mice [9, 19]. These researches indicate that polysaccharides have beneficial effects on metabolism and gut microbiota.

*WuGuChong*, a traditional Chinese medicine with function of improving digestion disorders, has been used to treat infantile malnutrition and fulminant dysentery, as reported in traditional Chinese medical journals such as *Compendium of Materia Medica* and *Materia Medica Companion* [20]. Recently, extracts from *WuGuChong* have been demonstrated to have antibacterial effects, promote wound healing, and inhibit the progression of diabetes and atherosclerosis [21–23]. However, little is known about the effects of *WuGuChong* on gut microbiota, obesity and its related complications. In the present study, the effects of polysaccharide extracted from *WuGuChong* (PEW) on HFD-induced obesity and obesity-related liver steatosis, insulin resistance and systemic inflammation, as well as gut integrity were evaluated. Through the gut microbiota analysis, the results indicated that PEW had a beneficial regulation on the composition of gut microbiota, suggesting a potential mechanism by which PEW attenuated obesity and its related metabolic syndromes. Our study demonstrates that PEW may be a potential bioactive ingredient for preventing obesity.

## Methods

### PEW preparation

The dried bodies of *WuGuChong* were purchased in a traditional Chinese medicine market and identified according to the standards in “Chinese Materia Medica Standards”. The PEW was isolated according to water extraction and alcohol precipitation methods as previous reported [24]. The Sevage method and DEAE iron-exchanged chromatography method were used for deproteinization and purification of polysaccharides. The PEW was characterized by GPC (gel permeation chromatogram) and HPLC (high performance liquid chromatography). The molecular weight of PEW was determined at 32.9 kDa, and its monosaccharide composition included rhamnose, glucose, mannose, galactose, arabinose, xylose, glucuronic acid, and galacturonic acid.

### Animal experiments

Eight-week old C57BL/6 male mice were obtained from the Experimental Animal Center in Dalian Medical University in China. After 1 week of acclimating to the new surroundings, the mice were randomly divided into three groups (*n* = 9 per group): The NCD (normal chow diet) group was conventionally fed a normal chow diet (3.656 kcal/g) containing 13.8% kcal fat (Jiangsu Xietong Pharmaceutical Bio-engineering Co., Ltd. China). The HFD group was fed a high fat diet (4.73 kcal/g) containing 45% kcal fat (D12451, Jiangsu Xietong Pharmaceutical Bio-engineering Co., Ltd. China). The PEW treatment group (HFD + PEW) was fed a high fat diet (D12451) and given PEW (300 mg/kg) by oral gavage once a day. The other two groups also had oral gavage with water once a day. The experimental treatment continued for 8 weeks. The mice were maintained in a light and climate controlled room at 25 ± 2 °C and 12 h light-dark cycle, with free access to water and different diets. Body weights and food intake were measured once a week. Liver and epididymal white adipose tissues (epi-WAT) were obtained and weighed immediately after treatment. The animal experimental protocols were approved by the ethics committee of Dalian Medical University (YJ-KY-SB-2019-83).

### Histopathological analysis

The epi-WAT tissues were routinely fixed in 4% paraformaldehyde, processed for hematoxylin and eosin (HE) staining, and examined using a microscope (Tokyo Olympus, Japan). The liver tissues were analyzed with Oil Red O staining according to the protocol described by Chang et al. [9].

### Biochemical analysis

Mice were fasted overnight after treatment, and blood samples were collected through the orbital sinus. The levels of total cholesterol (CHO), high and low density lipoprotein cholesterol (HDL-C and LDL-C), and triacylglycerol (TG) in the blood serum were measured using kits purchased from Nanjing Jiancheng Bioengineering Institute. Cytokine levels, including tumor necrosis factor-α (TNF-α), interleukin-6 (IL-6), and lipopolysaccharide (LPS), were analyzed using ELISA kits (Shanghai Lengton Biological Technology Co., Ltd. China).

### Glucose and insulin tolerance analysis

The intraperitoneal glucose and insulin tolerance tests (IPGTT and ITT) were conducted at weeks 7 and 8, respectively. In brief, mice were either fasted overnight or for 6 h, and then received an intraperitoneal injection of single-dose D-glucose (1.0 g/kg) or insulin (0.75 UI/kg; Novo Nordisk). Blood from the caudal vein was collected at 0, 15, 30, 60, and 120 min, respectively. A glucometer (Yuwell, Jiangsu, China) was used to measure blood glucose levels. Concentrations of fasting blood glucose and fasting serum insulin were measured, and the homeostasis model index of insulin resistance (HOMA-IR) was calculated according to the formula: HOMA-IR = insulin×glucose/22.5, as previously [25].

### Western blot analysis

Protein samples were extracted from the ileum epithelial tissue using RIPA lysis buffer, and then quantified using a BCA protein quantification kit (KeyGEN BioTECH, China). Proteins were loaded onto an 8% SDS-PAGE gel for electrophoresis, and then transferred onto PVDF membranes (Millipore, USA). After blocking with 5% skimmed milk, membranes were incubated with primary antibodies against ZO-1 (Zonula occludin-1, 1:1000; Proteintech), occludin (1:2000; Proteintech), and GAPDH (1:2000; Proteintech) over night at 4 °C, and then with secondary antibody for 2 h. Images were taken using ChemiDoc™ MP imaging system (Bio-Rad, America).

### Gene transcription analysis

Total RNA from ileum epithelial tissues was extracted using the TRIzol reagent (Invitrogen, USA) and reverse transcribed using the RevertAid First Strand cDNA Synthesis Kit (Thermo, USA). Real-time qPCR was conducted using the ABI 7500 Real-time PCR System (Applied Biosystems, USA). Values were normalized against β-actin and analyzed with the 2^−∆∆CT^ method. The real-time qPCR primers are listed in Table S1.

### Intestinal microbiota analysis

Fresh stool samples obtained from the colon immediately upon sacrificing the mice were frozen in liquid nitrogen and stored at − 80 °C. A stool DNA Kit (D4015, Omega, USA) was used to isolate the fecal genomic DNA. The V3-V4 region of 16S rRNA (338F-806R, F: ACTCCTACGGGAGGCAGCAG; R: GGACTACHVGGGTWTCTAAT) was amplified for identification. The DNA template was initially denatured at 98 °C for 30 s, then amplified for 35 cycles of denaturation (98 °C for 10 s), annealing (52 °C for 30 s), and extension (72 °C for 45 s), and finally extended at 72 °C for 10 min. The PCR products were purified by AMPure XT beads (Beckman Coulter Genomics, Danvers, MA, USA) and quantified by Qubit (Invitrogen, USA). The amplicon pools were prepared for sequencing and the size and quantity of the amplicon library were assessed on Agilent 2100 Bioanalyzer (Agilent, USA) and with the Library Quantification Kit for Illumina (Kapa Biosciences, Woburn, MA, USA), respectively. The PCR products were sequenced on an Illumina MiSeq platform (LC-Bio Technology Co., Ltd., Hang Zhou, China). Paired-end reads was assigned to samples based on their unique barcode and truncated by cutting off the barcode and primer sequence. Paired-end reads were merged using FLASH [26]. Quality filtering on the raw tags were performed under specific filtering conditions to obtain the high-quality clean tags according to the fqtrim (V 0.94). Chimeric sequences were filtered using Vsearch software (v2.3.4). Sequences with≥97% similarity were assigned to the same operational taxonomic units (OTUs) by Vsearch (v2.3.4) [27]. Representative sequences were chosen for each OTU, and taxonomic data were then assigned to each representative sequence using the RDP (Ribosomal Database Project) classifier. The differences of the dominant species in different groups, multiple sequence alignment were conducted using the mafft software (V 7.310) to study phylogenetic relationship of different OTUs. OTUs abundance information were normalized using a standard of sequence number corresponding to the sample with the least sequences. Alpha diversity is applied in analyzing complexity of species diversity for a sample through 5 indices, including Chao1, Observed species, Goods coverage, Shannon, and Simpson. All the indices in the samples were calculated with QIIME (Version 1.8.0). Beta diversity analysis was used to evaluate differences of samples in species complexity. For beta-diversity analysis, principal coordinate analysis (PCoA) and unweighted pair group method with arithmetic mean (UPGMA) clustering were performed by QIIME software (Version 1.8.0) [28]. The linear discriminant analysis (LDA) effect size (LEfSe) analysis was conducted for quantitative analysis of biomarkers in different groups [29]. Briefly, Kruskal-Wallis rank-sum test and Wilcoxon rank rank-sum test were used for LEfSe analysis (*P* < 0.05; LDA > 3.0), to identify the most differently abundant taxa.

### Statistical analysis

All data are presented as means±SD. The SPSS statistics 23.0 software (IBM, USA) was used for statistical analysis. Differences across groups were analyzed using one-way ANOVA followed by Tukey’s multiple comparison tests. Differences were considered as statistically significant if *P* < 0.05.

## Results

### PEW reduces body weight in HFD-treated mice

**Fig. 1:**
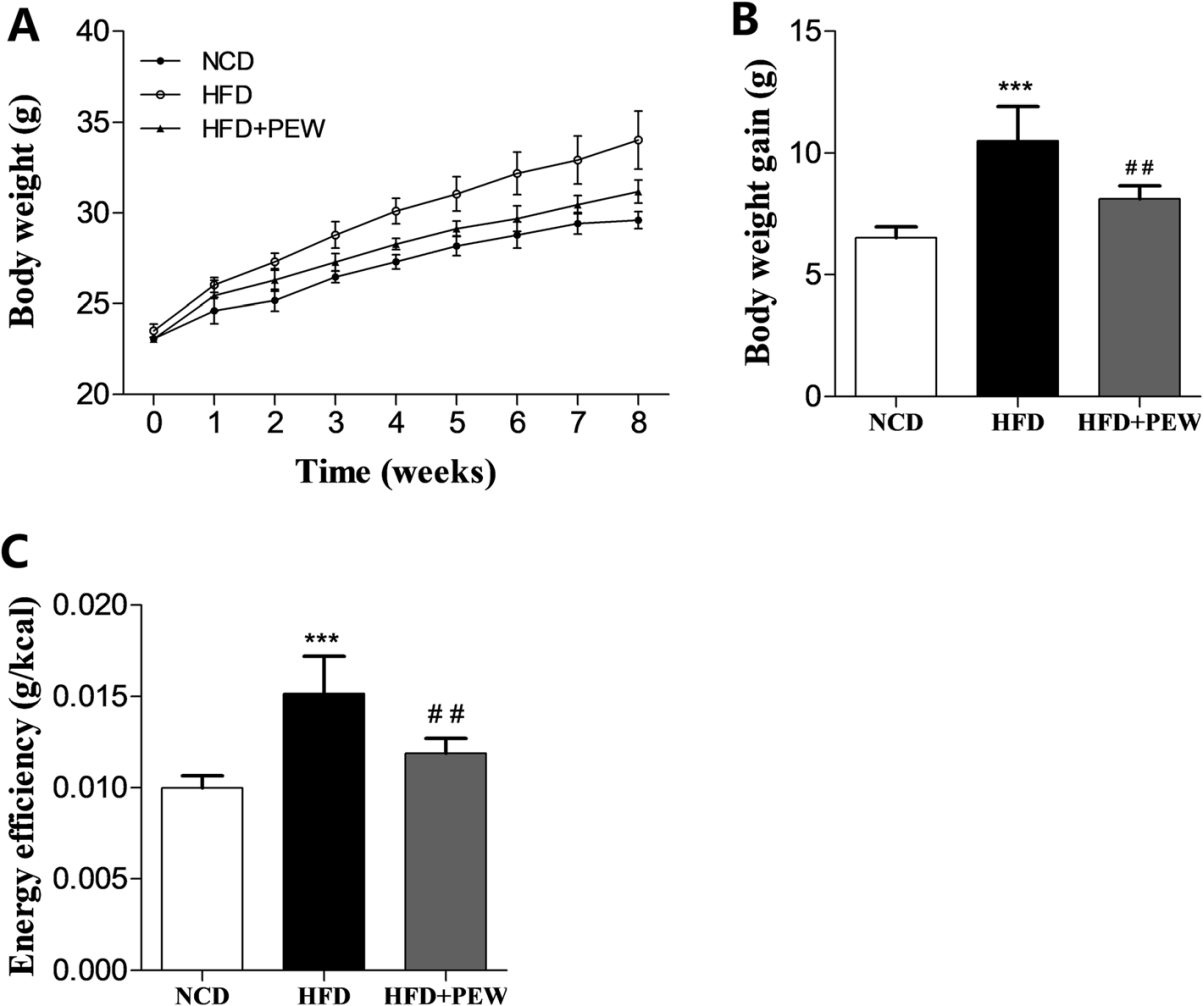
Effects of PEW on body weight (*n* = 5 for each group). **a** Body weight versus time analysis; **b** Body weight gain; **c** Energy efficiency, calculated according to the formula: body weight gain/energy intake. Data are presented as means±SD, and analyzed using the one-way ANOVA test with Tukey method. ^***^P < 0.001 compared with NCD; ^##^*P* < 0.05 compared with HFD

To determine the influence of PEW on body weight in HFD-treated mice, eight-week old C57BL/6 male mice were divided into three groups: NCD group, HFD group, HFD + PEW group. As expected, the HFD group exhibited increased body weight gain compared to the NCD group, which was reduced by PEW supplementation (Fig. 1a-b). Compared with the NCD group, the energy efficiency (weight gain divided by energy intake) in the HFD group was remarkably increased. Supplementation with PEW significantly decreased the energy efficiency in the HFD-fed mice (Fig. 1c), suggesting lower weight gain per kcal energy intake.

### PEW inhibits liver steatosis and adipose hypertrophy in HFD-treated mice

**Fig. 2:**
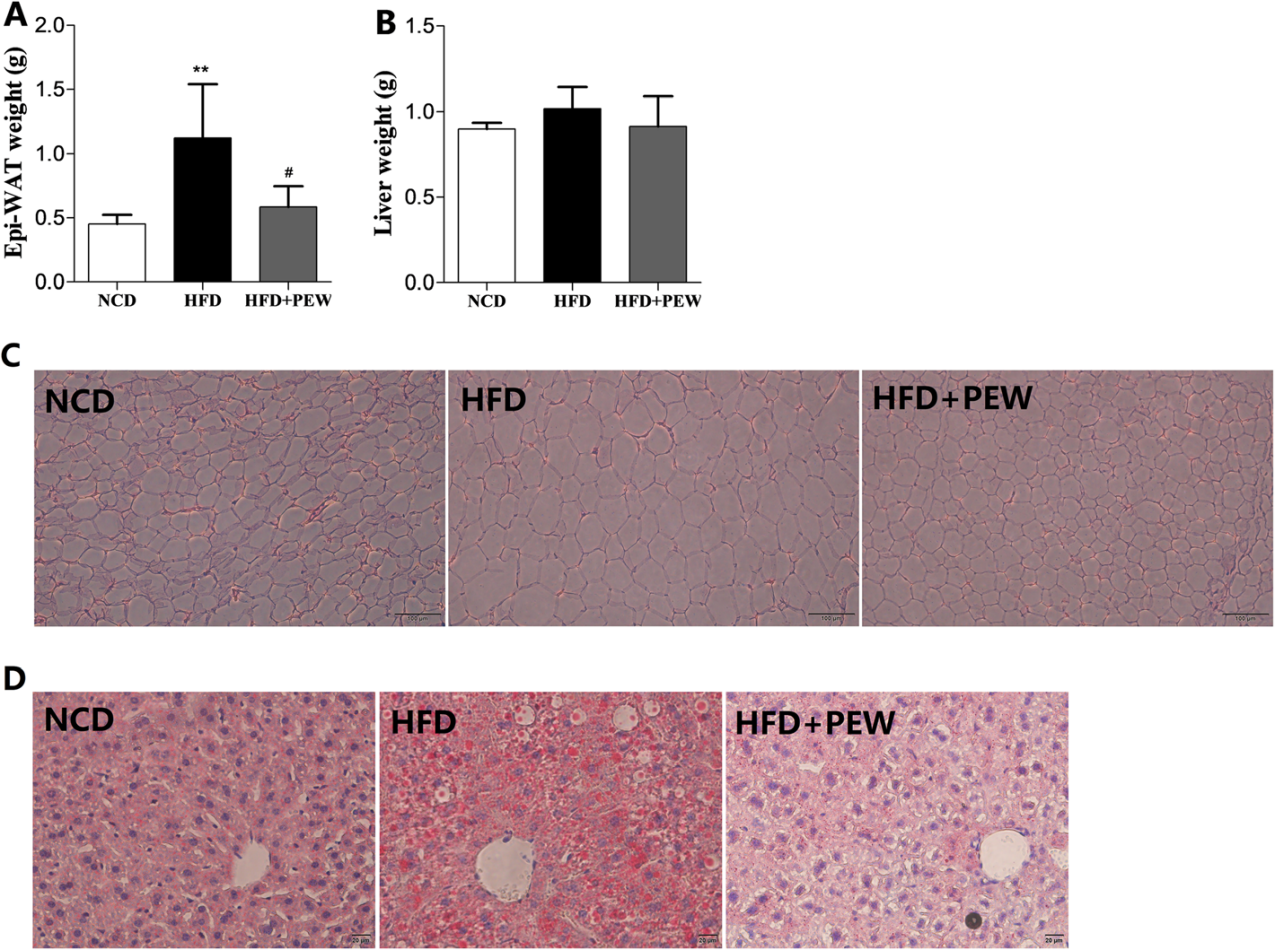
Effects of PEW on adipose hypertrophy and liver steatosis (*n* = 5 for each group). **a** epi-WAT weight; **b** Liver tissue weight. **c** HE staining of epi-WAT tissues (scale bar = 100 μm); **d** Oil red O staining of liver tissues (scale bar = 20 μm). Data are presented as means±SD, and analyzed using the one-way ANOVA test with Tukey method; ^**^P < 0.01 compared with NCD; ^#^*P* < 0.05 compared with HFD

Epi-WAT and liver weights were first measured to evaluate the effect of PEW on tissue changes. The epi-WAT weight was significantly induced by the HFD and reversed by PEW supplementation, while liver weight was not affected by HFD and PEW supplementation (Fig. 2a-b). Moreover, the HFD group exhibited larger adipocyte cells compared to the NCD group and PEW supplementation group (Fig. 2c), demonstrating that PEW prevents HFD-induced adipose hypertrophy. Compared to the NCD and PEW supplementation groups, fat accumulation in the liver was increased in the HFD group as indicated by Oil Red O staining (Fig. 2d), suggesting that PEW might attenuate liver steatosis.

### PEW attenuates serum lipids, cholesterol levels, insulin resistance, and glucose tolerance

**Fig. 3:**
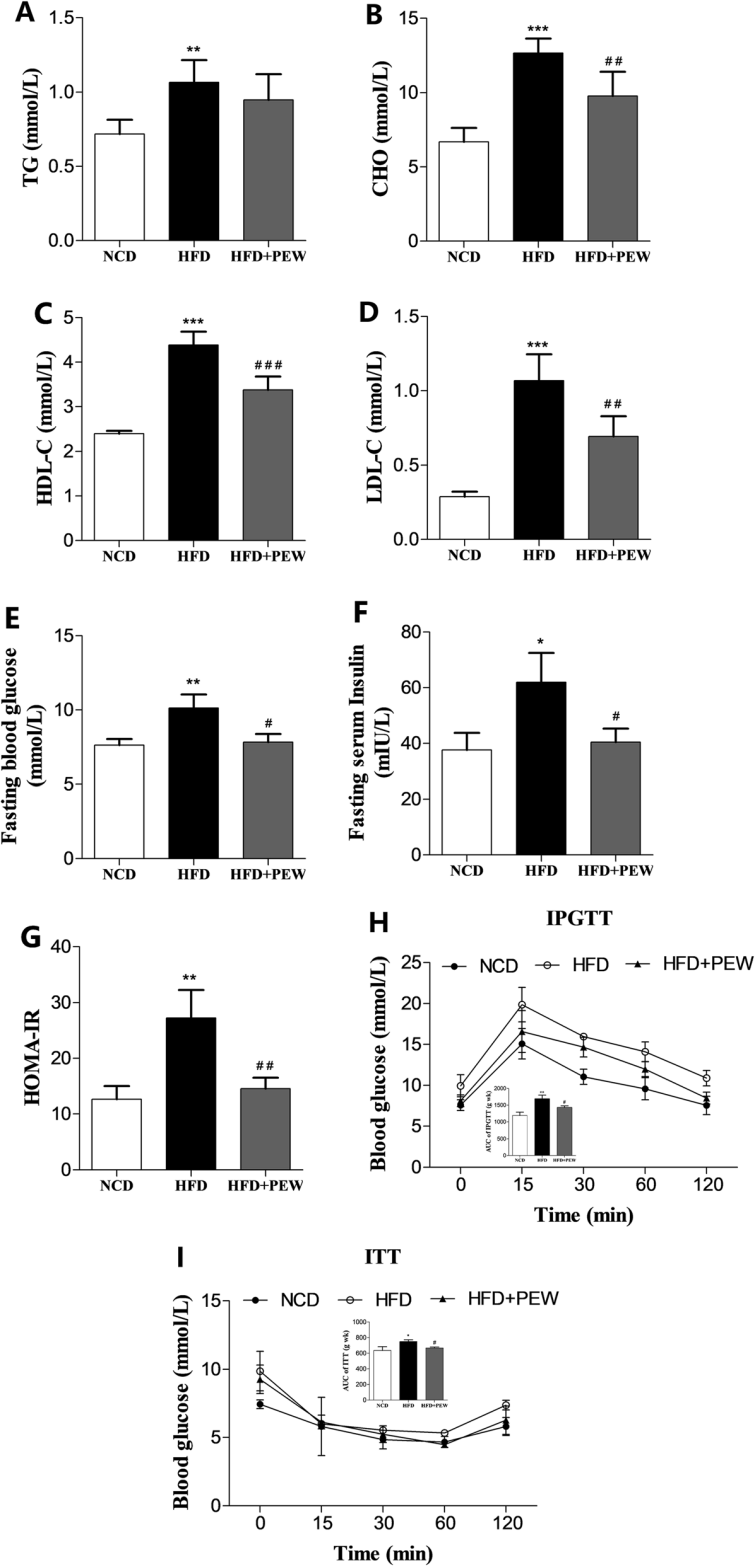
Effects of PEW on lipid concentration, glucose tolerance, and insulin resistance (*n* = 5 for each group). **a**-**d** TG (triacylglycerol), CHO (cholesterol), HDL-C (high density lipoprotein cholesterol), and LDL-C (low density lipoprotein cholesterol) levels in NCD, HFD, and HFD + PEW groups; **e**-**g** Fasting blood glucose, fasting serum insulin and HOMA-IR, calculated according to the formula: HOMA-IR = insulin×glucose/22.5. **h**-**i** Blood glucose versus time after intraperitoneal injection of D-glucose or insulin. The calculated AUC (inset) are also shown. Data are presented as means±SD, and analyzed using the one-way ANOVA test with Tukey method. ^*^P < 0.05, ^**^P < 0.01, ^***^P < 0.001 compared with NCD; ^#^*P* < 0.05, ^##^*P* < 0.01, ^###^*P* < 0.001 compared with HFD

To assess the ability of PEW to attenuate hypercholesterolemia and hyperlipidemia, we measured serum lipid concentrations in mice. The HFD group had a significant increase in TG, CHO, HDL-C, and LDL-C concentrations (48.2, 89.2, 86.9, and 270.8%, respectively) compared to the NCD group (Fig. 3a-d). Supplementation with PEW significantly decreased the CHO, HDL-C, and LDL-C concentrations (− 22.7, − 23.0, and − 35.2%, respectively) in the HFD-fed mice. However, the TG concentration was not significantly affected by PEW supplementation. These results suggested that PEW might attenuate hypercholesterolemia and hyperlipidemia in HFD-fed mice.

As obesity is closely correlated with insulin resistance and glucose tolerance [30], fasting blood glucose and fasting serum insulin was measured, and HOMA-IR was calculated according to the established formula. In addition, IPGTT and ITT tests were also performed in the present study. Supplementation with PEW maintained insulin sensitivity in HFD-fed mice, and improved fasting blood glucose, fasting serum insulin and HOMA-IR, which were closed to the control levels (Fig. 3e-g). As shown in Fig. 3h-i, compared with the NCD or PEW supplementation groups, the HFD group exhibited higher IPGTT, ITT, and AUC (area under curve) values, further suggesting that PEW supplementation significantly decreased the induced glucose tolerance and insulin resistance in HFD-treated mice.

### PEW prevents HFD-induced systemic inflammation

**Fig. 4:**
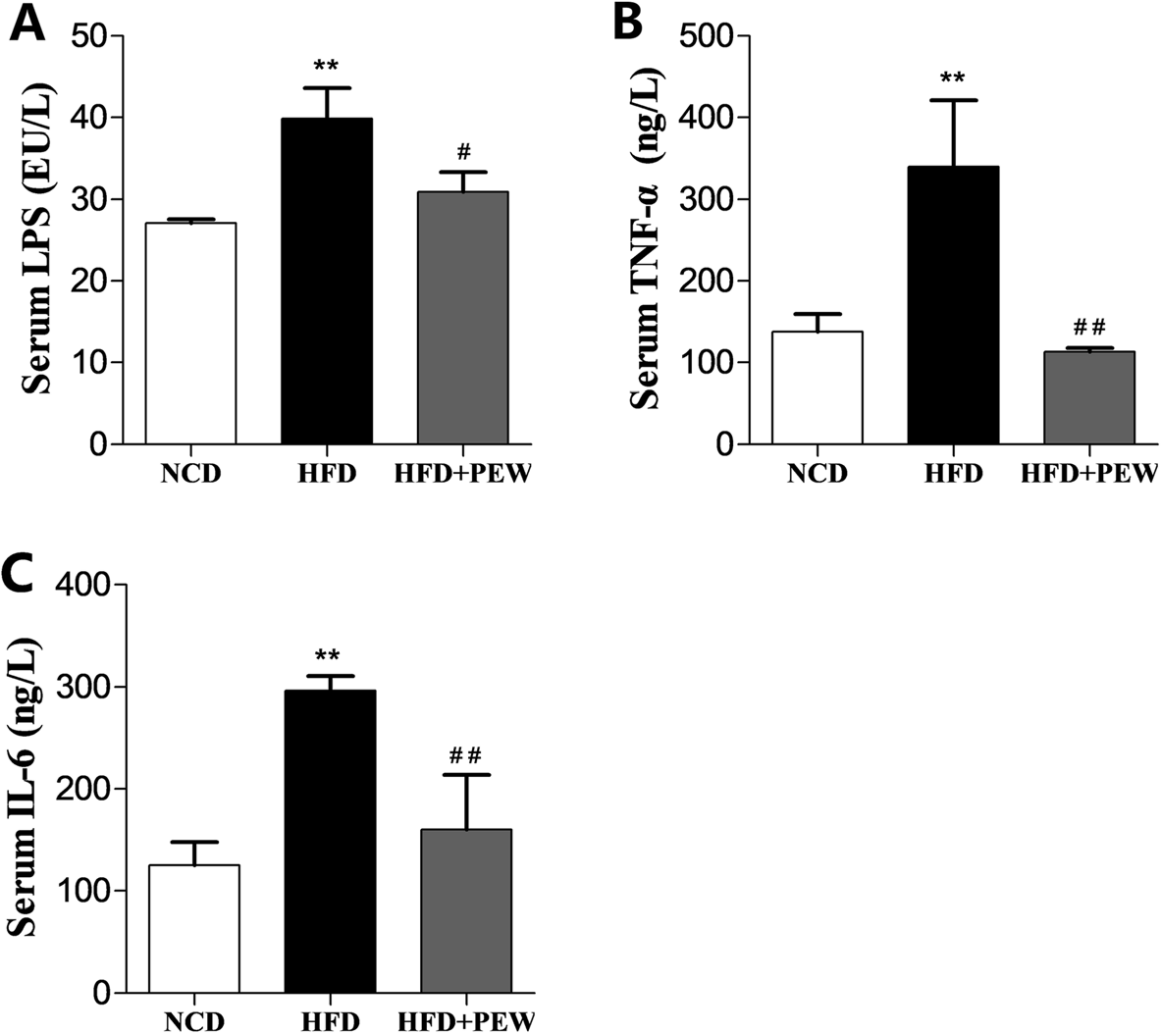
Effects of PEW on systemic inflammation (*n* = 5 for each group). **a** Serum level of endotoxins; **b** Serum level of TNF-α; **c** Serum level of IL-6. Data are presented as means±SD, and analyzed using the one-way ANOVA test with Tukey method. ^**^P < 0.01 compared with NCD; ^#^*P* < 0.05, ^##^*P* < 0.01 compared with HFD

It was previously reported that obesity and liver steatosis are accompanied by increased levels of serum endotoxins (LPS) and inflammatory cytokines [9, 31]. The effects of PEW on LPS release and cytokines secretion were examined in this study. As expected, compared with the NCD group, the HFD group displayed a significant increase in serum LPS, TNF-α, and IL-6 levels, which was reversed by PEW supplementation (Fig. 4a-c), suggesting that PEW might prevent HFD-induced endotoxemia and systemic inflammation.

### PEW maintains intestinal epithelium integrity

**Fig. 5:**
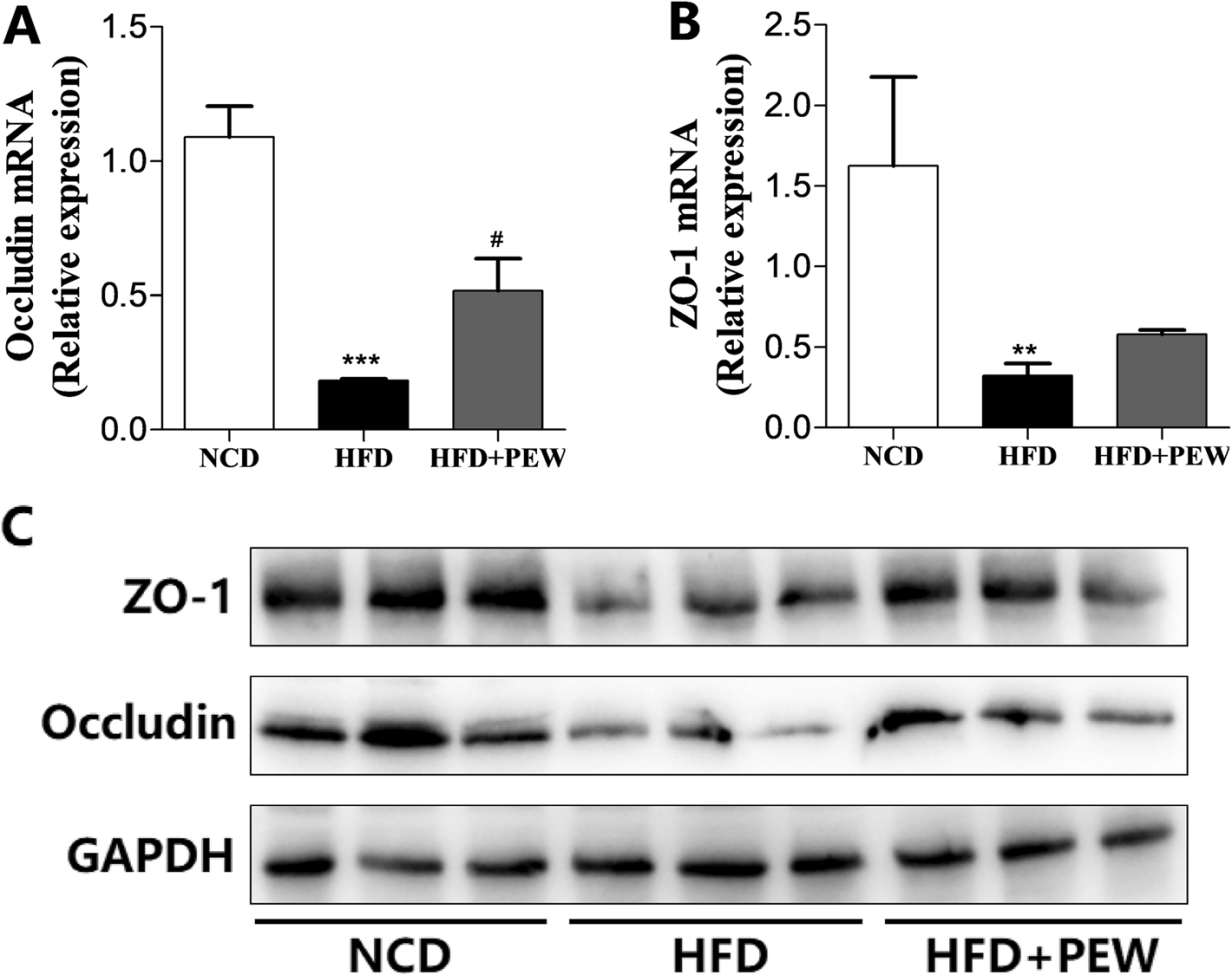
Relative expression of occludin and ZO-1 (*n* = 3 for each group). **a**-**b** RT-qPCR analysis of the relative mRNA levels of occludin and ZO-1; **c** Western blot analysis of occludin and ZO-1. Data are presented as means±SD, and analyzed using the one-way ANOVA test with Tukey method. ^**^P < 0.01, ^***^P < 0.001 compared with NCD; ^#^*P* < 0.05 compared with HFD

The effects of PEW on the integrity of the gut epithelium were evaluated by analyzing the expression of gut integrity biomarkers occludin and ZO-1 [32]. HFD strongly suppressed occludin and ZO-1 mRNA levels as identified by RT-PCR analysis (Fig. 5a-b). PEW supplementation significantly increased occludin levels but not ZO-1 in HFD-fed mice. However, the reduced occludin and ZO-1 protein expression in HFD-fed mice was recovered by PEW supplementation as shown by Western blot analysis (Fig. 5c). The results suggested that PEW supplementation maintained the gut integrity and barrier function.

### PEW alters the intestinal microbiota composition in HFD-fed mice

The intestinal microbiota composition was determined by sequencing the 16S rRNA (V3 + V4 region) using the Illumina MiSeq platform. Based on 97% identity level, OTU averages of 2511, 3561, and 3478 were respectively clustered in the NCD, HFD, and HFD + PEW groups. As shown in Fig. S1A, the OTUs in the HFD group were higher compared to the NCD group, while PEW supplementation did not significantly decrease the OTUs. The curves of OTU rank, Chao 1, Shannon, and Simpson are presented in Fig. S1B, C, D, and E, respectively. The indexes of Chao 1 (Fig. S1F), Shannon (Fig. S1G), and Simpson (Fig. S1H) were calculated. The curves of OTU rank and Chao 1 were consistent with the number of OTUs. No significant difference was found for Shannon and Simpson indexes, suggesting that there was no difference in richness and diversity of intestinal microbiota between the three groups.

**Fig. 6:**
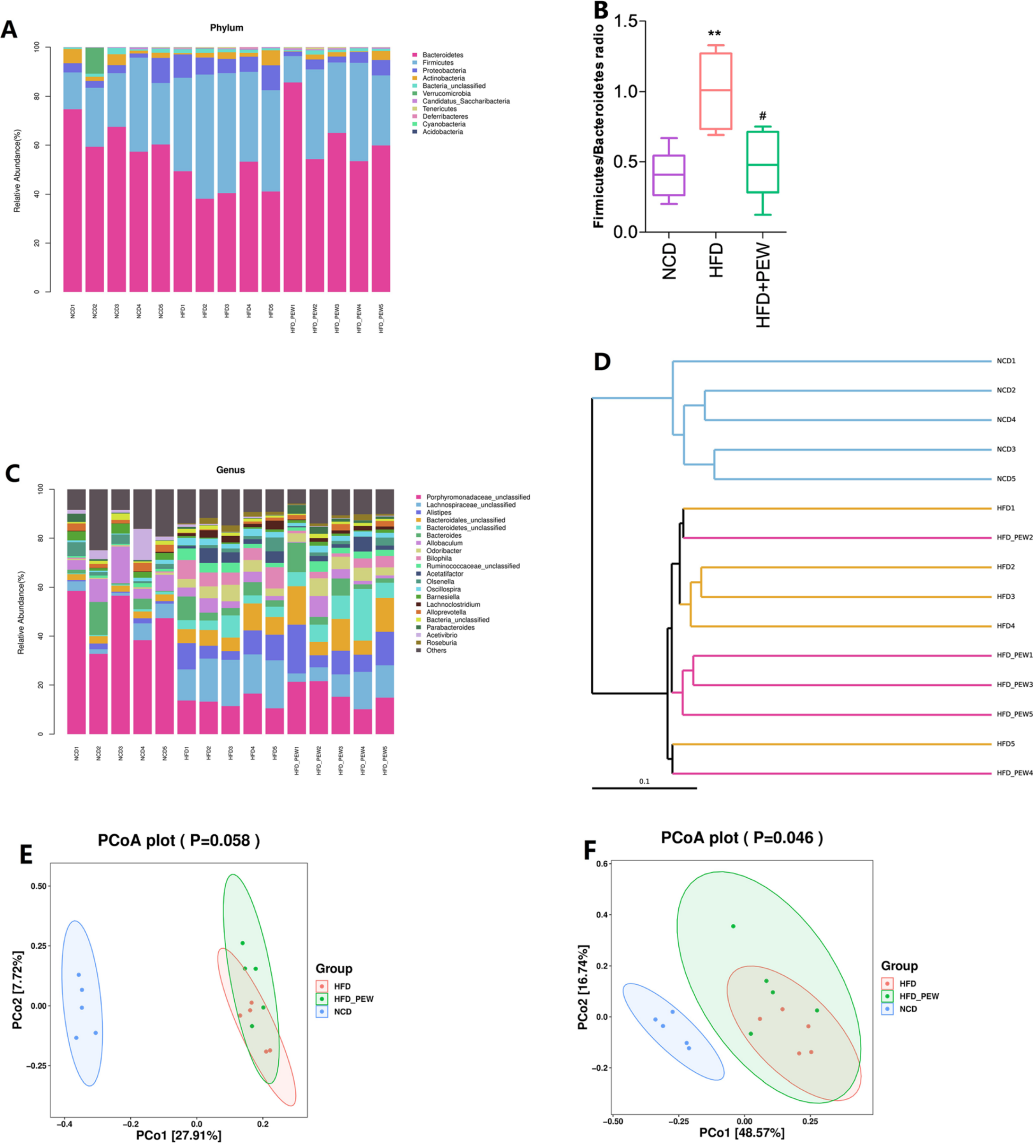
The variation of gut microbiota at phylum and genus levels (*n* = 5 for each group). **a** Relative abundance of bacteria at the phylum level; **b** The *Firmicutes* to *Bacteroidetes* ratio in NCD, HFD, and HFD + PEW groups; **c** Relative abundance of bacteria at the genus level; **d** Unweighted UPGMA of all samples; **e**-**f** Plots of unweighted and weighted UniFrac-based PCoA. Data are presented as means±SD, and analyzed using the one-way ANOVA test with Tukey method. ^**^P < 0.01 compared with NCD; ^#^*P* < 0.05 compared with HFD

As shown in Fig. 6a, the relative decreased abundance of *Bacteroidetes* and increased abundance of *Firmicutes* and *Proteobacteria* were observed in the HFD group in the phylum level, and the F/B ratio was also increased in the HFD group compared to the NCD group. PEW supplementation restored the levels of *Bacteroidetes*, *Firmicutes,* and *Proteobacteria*, and significantly decreased the F/B ratio (Fig. 6b). The difference of genus level among each group was further investigated, and the results were consistent with the phylum level. PEW supplementation significantly increased the relative abundance of *Parabacteroides* belonging to the *Bacteroidetes* phylum, and significantly decreased the relative abundance of *Ihubacter*, *Lachnospiraceae_unclassified*, and *Lachnostridium* belonging to the *Firmicutes* phylum, as well as *Bilophila* belonging to the *Proteobacteria* phylum in HFD-treated mice (Fig. 6c). In addition, unweighted unifrac cluster based UPGMA and the unweighted and weighted unifrac distance based PCoA were performed to investigate gut microbiota structural changes. As shown in Fig. 6d-f, all three groups exhibited distinctive microbiota profiles. Furthermore, the HFD + PEW group and HFD group showed a similar microbiota structure.

**Fig. 7:**
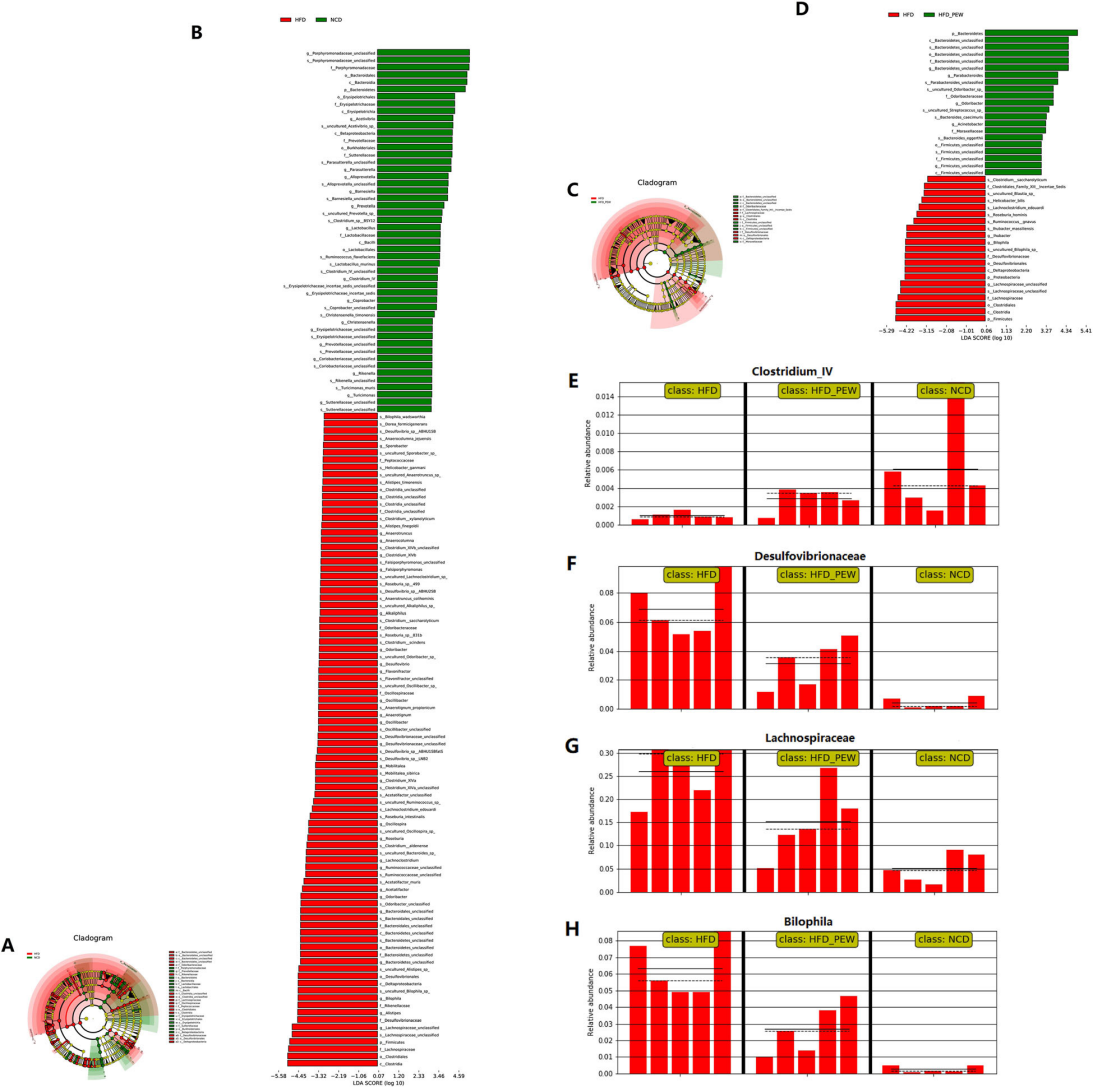
LEfSe results. **a**-**b** Cladogram showing the phylogenetic relationships of bacteria taxa and LDA scores between HFD and NCD group; **c**-**d** Cladogram showing the phylogenetic relationships of bacteria taxa and LDA scores between HFD and HFD + PEW group; **e-h** Relative abundance of *Clostridium_IV*, *Desulfovibrionaceae*, *Lachnospiraceae*, and *Bilophila* in gut microbiota, respectively

The biomarkers in the gut microbiota sequences were analyzed using the LEfSe method. Compared with the HFD group, the successive circles and bar graph showed that 50 phylotypes were higher and 90 were lower in the NCD group, and 21 phylotypes were higher and 21 were lower in the HFD + PEW group (Fig. 7a-d). The results indicate that PEW supplementation significantly increased the abundance of beneficial phylotypes *Bacteroidetes*, and decreased the abundance of pathogenic phylotypes *Firmicutes* and *Proteobacteria* in HFD-treated mice. As shown in Fig. 7e, PEW supplementation induced an increase in *Clostridium_IV*, which had a potential probiotic effect [33]. The abundance of pathogenic bacteria, including *Desulfovibrionaceae*, *Lachnospiraceae*, and *Bilophila* [34–36], were reduced after PEW supplementation in HFD-treated mice (Fig. 7f-h). These results indicated that PEW supplementation might prevent HFD-induced intestinal microbiota dysbiosis.

## Discussion

Gut microbiota is closely associated with obesity and its related metabolic disorders [7]. Recent research data show that polysaccharides extracted from natural products prevent HFD-induced obesity and its related complications by modulating the composition of gut microbiota [9, 19]. However, the effects of PEW on obesity and gut microbiota has not been investigated. In the study, we investigated the effects of PEW supplementation for 8 weeks on HFD-induced obesity and gut microbiota. The results demonstrate that PEW could prevent HFD-induced obesity, and its related liver steatosis, insulin resistance and systemic inflammation by modulating the composition of gut microbiota. In addition, PEW supplementation could also maintain the intestinal epithelium integrity.

In the present study, PEW supplementation significantly prevents body weight gain, adipose hypertrophy in HFD-fed mice (Fig. 1a-b and Fig. 2a, c). As previously reported, the composition of gut microbiota in obese and lean individuals was significantly different [8]. In the gut microbiota of obese models, increased abundances of *Firmicutes* and decreased abundances of *Bacteroidetes* were observed [9, 37]. Our results show that HFD increases the abundance of *Firmicutes* and decreases the abundance of *Bacteroidetes*. Consistent with the Chang’s research [9], PEW supplementation significantly reverses the changes, and decreases the increased F/B ratio in HFD-fed mice to approximately the control levels (Fig. 6a-b), which may contribute to its anti-obesity effects. Additionally, at the phylum level, endotoxin-bearing *Proteobacteria* is also decreased by PEW supplementation. Obesity is often accompanied by fat accumulation in the viscera, especially in liver, and closely associated with chronic systemic inflammation, which may induce many chronic diseases [38]. As expected, PEW supplementation attenuates liver steatosis induced by HFD in the study (Fig. 2d). Previous evidence indicated that significantly increased inflammatory cytokine TNF-α and IL-6 levels were observed in obese individuals [39]. The pro-inflammatory molecules can affect the host metabolic process, as TNF-α was reported to decrease the insulin sensitivity and increase lipolysis in adipocytes, as well as IL-6 was found to contribute to hypertriglyceridemia [40, 41]. In our study, serum levels of TNF-α and IL-6, lipids, cholesterol, glucose and insulin increased significantly in HFD group (Fig. 3 and Fig. 4b-c). However, PEW supplementation significantly reduces systemic inflammation, insulin resistance, hypercholesterolemia and hyperlipidemia in HFD-fed mice. The results suggest that PEW improves obesity-related metabolic disorders by modulating systemic inflammation.

Accumulating evidence suggests that gut microbiota is closely linked to host systemic inflammation, which is a feature of obesity [39, 42]. Significantly increased inflammatory cytokine TNF-α and IL-6, as well as LPS, all markers of systemic inflammation, were observed in obese models as previous report [43], and decreased by PEW supplementation (Fig. 4a-c). Gut microbiota dysbiosis, which contributes to LPS production, is the potential mechanism for systemic inflammation induced by HFD. Endotoxins, known as LPS, which induced the release of pro-inflammatory cytokine, were predominantly derived from Gram-negative bacteria in intestinal microbiota [44, 45]. In the present study, the relative abundance of endotoxin-producing bacteria *Desulfovibrionaceae* is increased in HFD group, and reversed by PEW supplementation (Fig. 7f). The results are in agreement with previous research, which demonstrated that the relative abundance of *Desulfovibrionaceae* was decreased by dietary intervention in obese individuals [34]. The relative abundance of *Bilophila* has been demonstrated to be positively related to obesity and inflammation [36]. In the present study, the relative abundance of *Bilophila* is increased by HFD, and restored by PEW supplementation (Fig. 7h), which is consistent with the previous report. Recent research observed that the relative abundance of *Lachnospiraceae* was increased in high-fat/high-sucrose (HFHS)-fed mice [35]. Here, PEW supplementation significantly decreases the relative abundance of *Lachnospiraceae*, which is increased by HFD (Fig. 7g). In addition, *Clostridium_IV*, belonging to *Clostridium* clusters, which lacks prominent toxins and virulence factors, has potential probiotic effects. Meanwhile, *Clostridium_IV* was also reported to regulate host fatty acid metabolism, immune function and ameliorated colitis [33]. Our study demonstrates that PEW supplementation enhances the relative abundance of *Clostridium_IV* in HFD-treated mice (Fig. 7e). The results suggest that the anti-obesity and anti-inflammation effects of PEW may be due to modulation of the gut microbiota composition.

Previous study reported that HFD-induced gut microbiota dysbiosis contributed to the damage of intestinal epithelium integrity, and the release of LPS into circulation, resulting in insulin resistance, systemic inflammation and obesity [9]. Otherwise, gut microbiota could also help strengthen mucosal defense by promoting epithelial renewal and immune maturation, as well as decreasing intestinal permeability [46, 47]. In the study, PEW supplementation increases the expression of occludin and ZO-1, which are decreased by HFD (Fig. 5), resulting in maintaining intestinal epithelium integrity. This is consistent with the previous study, which reported that increased expression of occludin and ZO-1, along with modulation of the gut microbiota composition, contributed to strengthen the gut integrity and barrier function [48]. The results suggest that PEW may improve obesity-related metabolic disorders by modulating gut microbiota and maintaining intestinal epithelium integrity.

In conclusion, our results show that PEW supplementation prevents HFD-induced obesity and its related metabolic disorders, including liver steatosis, insulin resistance and systemic inflammation, by modulating gut microbiota composition and maintaining intestinal epithelium integrity. PEW supplementation decreases *Firmicutes* to *Bacteroidetes* ratio and the relative abundance of *Proteobacteria* in HFD-fed mice, contributing to the beneficial effects against obesity and its related disorders. Our study suggests that PEW may be used as a bioactive ingredient to prevent obesity. However, the detailed mechanisms need to be further investigated.

## Supplementary information

**Additional file 1: Table S1.** Primers used in this study.

**Additional file 2: Figure S1.** The characteristics of gut microbiota. (A) OTU clusters of gut microbiota; (B-E) OTU rank curves, Chao 1 curves, Shannon curves, and Simpson curves of gut microbiota, respectively; (F-H) Chao 1, Shannon, and Simpson indexes of gut microbiota, respectively. Data are presented as means and standard deviation, and analyzed using the one-way ANOVA test. ^**^P < 0.01 compared with NCD.

## Data Availability

The datasets used and/or analyzed during the current study are available from the corresponding author on reasonable request.

## Abbreviations

CHO: Total cholesterol
epi-WAT: Epididymal white adipose tissues
GPC: Gel permeation chromatogram
HE: Hematoxylin and eosin
HFD: High-fat diet
IPGTT: Intraperitoneal glucose test
ITT: Insulin tolerance test
LDA: Linear discriminant analysis
NCD: Normal chow diet
OTU: Operational taxonomic unit
PEW: Polysaccharide extracted from *WuGuChong*
ZO-1: Zonula occludin-1
