# Supplementary material for: Polysaccharide extracted from WuGuChong reduces high-fat diet-induced obesity in mice by regulating the composition of intestinal microbiota
Source: Nutr Metab (Lond). 2020 Mar 30;17:27. doi: 10.1186/s12986-020-00442-2 (PMC7106597; doi:10.1186/s12986-020-00442-2)
Supplement: Supplementary file 1 — Additional file 1: Table S1. Primers used in this study. [file 12986_2020_442_MOESM1_ESM.docx]

**Table S1.** Primers used in this study

| Target Gene | Primer sequence |
| --- | --- |
| Occludin F | TTTCCTTAGGCGACAGCG |
| Occludin R | CCATCTTTCTTCGGGTTT |
| ZO-1 F | TGCCTCGAACCTCTACTC |
| ZO-1 R | GTGGTGGAACTTGCTCAT |
| β-actin F | CTGTGCCCATCTACGAGGGCTAT |
| β-actin R | TTTGATGTCACGCACGATTTCC |
