# Supplementary material for: Polysaccharide extracted from WuGuChong reduces high-fat diet-induced obesity in mice by regulating the composition of intestinal microbiota
Source: Nutr Metab (Lond). 2020 Mar 30;17:27. doi: 10.1186/s12986-020-00442-2 (PMC7106597; doi:10.1186/s12986-020-00442-2)
Supplement: Supplementary file 2 — Additional file 2: Figure S1. The characteristics of gut microbiota. (A) OTU clusters of gut microbiota; (B-E) OTU rank curves, Chao 1 curves, Shannon curves, and Simpson curves of gut microbiota, respectively; (F-H) Chao 1, Shannon, and Simpson indexes of gut microbiota, respectively. Data are presented as means and standard deviation, and analyzed using the one-way ANOVA test. **P < 0.01 compared with NCD. [file 12986_2020_442_MOESM2_ESM.docx]

**Figure S1**


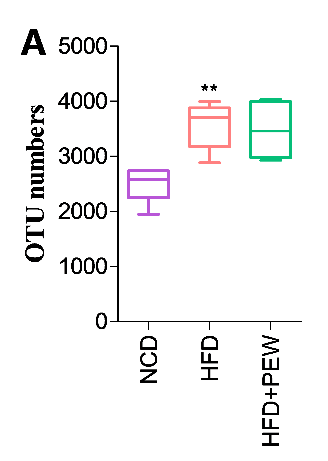

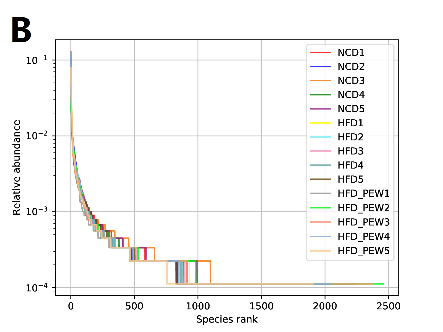

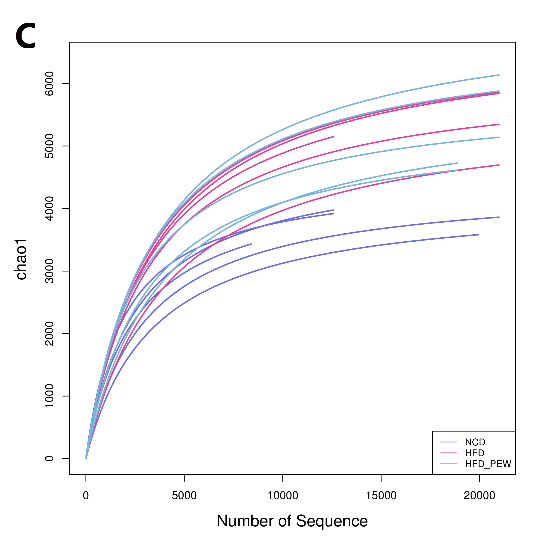

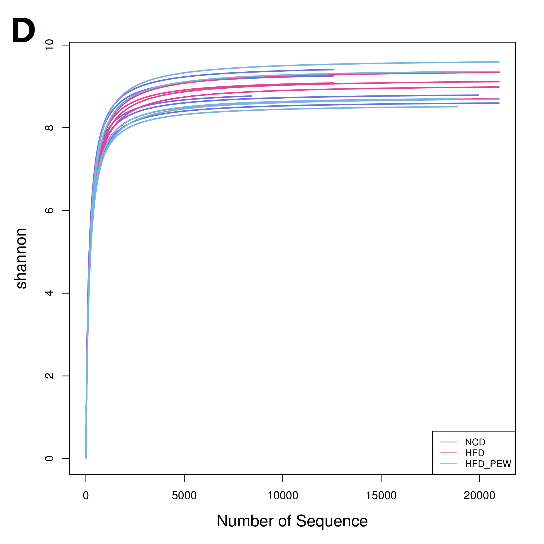

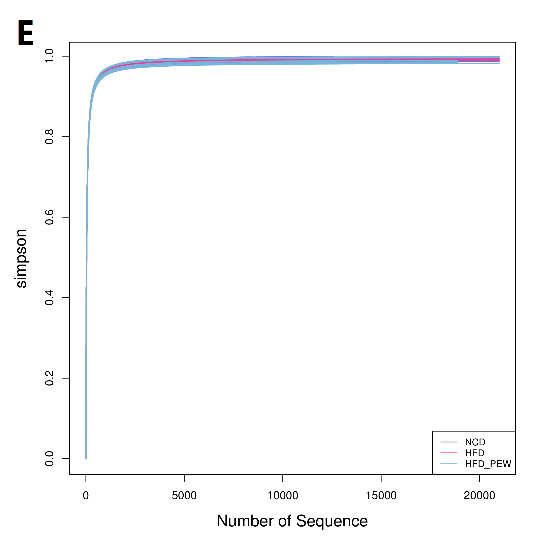

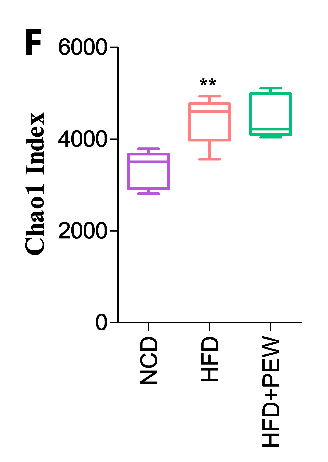

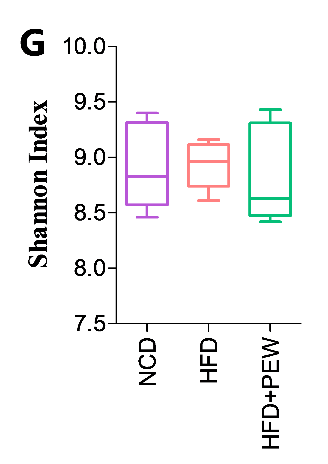

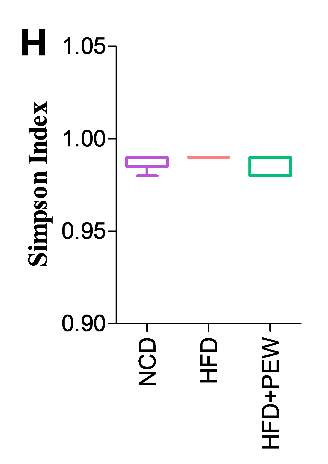


**Figure S1.** The characteristics of gut microbiota. (A) OTU clusters of gut microbiota; (B-E) OTU rank curves, Chao 1 curves, Shannon curves, and Simpson curves of gut microbiota, respectively; (F-H) Chao 1, Shannon, and Simpson indexes of gut microbiota, respectively. Data are presented as means and standard deviation, and analyzed using the one-way ANOVA test. ^**^P < 0.01 compared with NCD.
